# Superfluorinated, Highly Water-Soluble Polyphosphazenes as Potential ^19^F Magnetic Resonance Imaging (MRI) Contrast Agents

**DOI:** 10.3390/jfb15020040

**Published:** 2024-02-10

**Authors:** Paul Strasser, Verena Schinegger, Joachim Friske, Oliver Brüggemann, Thomas H. Helbich, Ian Teasdale, Irena Pashkunova-Martic

**Affiliations:** 1 Institute of Polymer Chemistry, Johannes Kepler University Linz, Altenberger Straße 69, 4040 Linz, Austria; 2 Department of Biomedical Imaging and Image-Guided Therapy, Division of Structural and Molecular Preclinical Imaging, Medical University of Vienna and General Hospital of Vienna, 18-20 Währinger Gürtel, 1090 Vienna, Austria

**Keywords:** gadolinium-free contrast agents, ^19^F-MRI, superfluorinated polymers, biodegradable polymers, polyphosphazene

## Abstract

“Hot spot” ^19^F magnetic resonance imaging (MRI) has garnered significant attention recently for its ability to image various disease markers quantitatively. Unlike conventional gadolinium-based MRI contrast agents, which rely on proton signal modulation, ^19^F-MRI’s direct detection has a unique advantage in vivo, as the human body exhibits a negligible background ^19^F-signal. However, existing perfluorocarbon (PFC) or PFC-based contrast materials suffer from several limitations, including low longitudinal relaxation rates and relatively low imaging efficiency. Hence, we designed a macromolecular contrast agent featuring a high number of magnetically equivalent ^19^F-nuclei in a single macromolecule, adequate fluorine nucleus mobility, and excellent water solubility. This design utilizes superfluorinated polyphosphazene (PPz) polymers as the ^19^F-source; these are modified with sodium mercaptoethanesulfonate (MESNa) to achieve water solubility exceeding 360 mg/mL, which is a similar solubility to that of sodium chloride. We observed substantial signal enhancement in MRI with these novel macromolecular carriers compared to non-enhanced surroundings and aqueous trifluoroacetic acid (TFA) used as a positive control. In conclusion, these novel water-soluble macromolecular carriers represent a promising platform for future MRI contrast agents.

## 1. Introduction

Classical magnetic resonance imaging (MRI) is an unprecedented clinical imaging modality used to diagnose and monitor numerous pathologies in vivo. To improve MR sensitivity and specificity, the era of contrast-enhanced MRI (CE-MRI) has offered groundbreaking insights into the delineation of benign and malignant lesions covering all parts of the body, assessment of perfusion [1], evaluation of response to anti-angiogenic therapies [2,3,4,5,6,7,8], as well as the prediction of outcomes after chemotherapy. Contrast agents (CAs), which are used in a clinical setting and which improve diagnostic accuracy, are exclusively small, hydrophilic gadolinium(III) (Gd(III))-based chelates. The currently clinically available Gd(III) agents are specifically designed to move through patients passively, with little or no interaction with proteins or cells, and provide superb contrast to differentiate anatomical structures and characterize pathophysiology. However, this occurs in a nonspecific manner (by interactions with protons present in the living body) [9]. Furthermore, Gd(III)-based contrast agents are contraindicated in patients with acute and chronic renal insufficiency [10]. In addition, recent studies have reported the deposition of free Gd(III) in neural tissue, bones and muscles in patients who have undergone systemic CE-MRI [11,12]. Thus, there is an urgent clinical need for alternative MRI contrast agents that are acceptable in the context of renal insufficiency.

Non-proton or X-nuclei MRI is based on the detection of the nuclei of other atoms (X-nuclei) in the body, such as sodium (^23^Na), phosphorus (^31^P), chlorine (^35^Cl), potassium (^39^K), deuterium (^2^H), oxygen (^17^O), lithium (^7^Li), and fluorine (^19^F), using modified software and hardware. In addition, X-nuclei MRI can provide fundamental, new metabolic information related to cellular energetic metabolism and ion homeostasis in tissues that cannot be assessed using standard proton MRI. In particular, ^19^F has numerous attractive properties, such as 100% natural abundance and a negligible ^19^F background signal in tissues, prompting extensive research into ^19^F-MRI probes [13]. In addition, it allows for quantitative detection of distinct molecular targets in vivo due to the broad chemical shift range in nuclear magnetic resonance (NMR) of various organofluorine species applied as signal enhancers for ^19^F-MRI [14]. Thereby, ^19^F-MRI is an emerging modality, predominantly for spectral molecular imaging and cell tracking. However, the sensitivity of ^19^F-MRI remains a challenge, as the local ^19^F concentration cannot come close to the ^1^H content present in the human body. Thus, ^19^F-MRI CAs that provide high fluorine content and good water solubility are required to reach detectable ^19^F concentrations in vivo. Several fluorinated probes for in vivo ^19^F-MRI have been reported to date, most of them based on liquid perfluorocarbons (PFCs) and perfluoropolyether (PFPE) nanoemulsions used for quantitative imaging of transplanted cells, angiogenesis or inflammation [15]. Nevertheless, these investigated current exogenous probes, such as PFCs and PFPEs, have been solely used for cell tracking and their in vivo application is strongly limited due to their hydrophobicity [15,16]. Furthermore, the sensitivity of ^19^F-MRI remains a challenge, as the local ^19^F concentration cannot approach the ^1^H content present in the human body by far. Thus, ^19^F-MRI CAs that provide high fluorine content are required to reach detectable ^19^F concentrations in vivo. Furthermore, PFC-nanoparticles have been investigated for the mapping of tumor oxygenation [16]. However, these formulations require distinctly high concentrations to enable detection by MRI. The need for auxiliary surfactants and the multiple resonance frequencies account for additional shortcomings [17,18,19]. Alternative approaches employ the design of specific fluorinated monomers, incorporated in macromolecular structures by controlled polymerization methods such as RAFT [20,21] or ROMP [19]. Thereby, co-polymerization or post-polymerization reactions provide additional functionality or water solubility.

Poly(organo)phosphazenes (PPz) represent a versatile family of hybrid polymers that combine organic and inorganic components [22,23]. These polymers feature a highly flexible inorganic backbone composed of [NPR_2_]_n_ repeat units. They are of high interest in the field of biomedicine, with potential applications such as stent coatings, drug and protein delivery systems, and vaccine adjuvants [24,25]. The controlled synthesis of these polymers, primarily through living cationic polymerization methods, allows for the creation of polymers with precisely controlled length and a narrow range of characteristics (referred to as Ð) [26,27]. Subsequently, various modification reactions can be employed after polymerization to fine-tune the functionality, degradability, and overall architecture of PPz. Indeed, trifluoroethoxypolyphosphazene has gained FDA approval, for example, in the form of so-called polyzene-F nanocoatings on embolic spheres and coronary stents, and is progressing to advanced clinical trials in Europe; the same has occurred for microbeads used in cancer therapy [28]. Concurrently, there has been significant progress in the development of degradable polyphosphazenes for use as versatile drug and vaccine carriers [25,29,30,31]. These polyphosphazenes possess the unique qualities of degradability, high water solubility, and multifunctionality. In this study, we report for the first time on the synthesis and evaluation of novel superfluorinated yet highly water-soluble PPz as a tunable platform for the design of next-generation Gd(III)-free MRI contrast agents.

## 2. Materials and Methods

Chemicals were purchased from different commercial sources and used as received, unless stated otherwise. Deuterium oxide (D_2_O) and deuterated acetone ((CD_3_)_2_CO) were purchased from Deutero, anhydrous LiBr from Alfa Aesar and HPLC-grade dimethylformamide (DMF) from Fisher Chemicals (Schwerte, Germany). Trifluoroacetic acid (TFA), ≥99% p.a., phosphate-buffered saline (PBS) and fetal bovine serum (FBS) used in ^19^F-MRI, as well as 2,2,2-trifluoroethanol, 4-(diphenylphosphino)styrene, 2,2-dimethoxy-2-phenylacetophenone (DMPA), hexachloroethane, sodium 2-mercaptoethanesulfonate (MESNa) and propargyl alcohol were bought from Sigma Aldrich (St. Louis, MO, USA) and NaH from Sigma Aldrich or BLDpharm (Shanghai, China). Anhydrous dichloromethane (DCM) and anhydrous tetrahydrofuran (THF) were obtained from Thermo Scientific (Waltham, MA, USA) and ethanol (EtOH), methanol (MeOH) and n-heptane from VWR (Radnor, IN, USA). PBS (phosphate-buffered saline) buffers were prepared from PBS tablets from VWR and the pH was adjusted accordingly with NaOH or HCl. The polyphosphazene monomer, Cl_3_PNSiMe_3_, was synthesized according to the literature and routine laboratory procedures [31,32].

Nuclear magnetic resonance (NMR) spectra were recorded on a Bruker Avance III 300 MHz spectrometer in deuterated solvents at 25 °C. IR (infra-red) spectra were measured with a PerkinElmer 100 Series FT-IR spectrometer equipped with ATR using a scan number of 128. Size exclusion chromatography (SEC) in organic media was performed on a Viscotek GPCmax instrument (Malvern Panalytical, Malvern, UK) using a PFG column from Polymer Standard Service GmbH (PSS) (Mainz, Germany) (300 mm × 8 mm, 5 µm particle size). The samples were filtered through 0.2 µm PTFE syringe filters prior to injection and eluted with DMF containing 10 mM LiBr as the mobile phase at a flow rate of 0.75 mL min^−1^ at 60 °C. The molecular weights were measured using a conventional calibration of the refractive index detector, calibrated with polystyrene standards from Polymer Standard Service GmbH (PSS). Dynamic light scattering (DLS) measurements were performed on a Malvern Zetasizer Nano ZSP at 25 °C at a fixed scattering angle of 173°. The sample solutions (0.2 mg mL^−1^) were prepared in PBS pH 7.4, sonicated for 10 min and filtered through 0.2 µm nylon syringe filters prior to the measurement. The zeta potential was determined using a Zetasizer Nano-ZS from Malvern (Malvern Panalytical, Malvern, UK) in nanopure water as dispersant (viscosity = 0.8872 cP and refractive index, RI = 1.330), calibrated with a Zeta Potential transfer standard (−40 ± 5.8 mV). Sample preparation was analogous to DLS measurements with a sample concentration of 0.5 mg mL^−1^. Thiol–yne reactions were performed at 5 °C in a photochemical reactor from Rayonet (Southern New England Ultraviolet, Branford, CT, USA) using UV light with a wavelength of 365 nm. Dialysis was carried out using Spectrum Spectra/Por RC membrane tubing with 1 kDa molecular weight cut-offs (MWCOs). EtOH used as a solvent was recycled by distillation. Samples were lyophilized with an Alpha 1–2 LDplus freeze-drying system from Christ.

All magnetic resonance imaging (MRI) and magnetic resonance spectroscopy (MRS) measurements were performed using a 9.4 T (BioSpec 94/30 USR, Avance Neo, Bruker, Ettlingen, Germany) horizontal bore small animal MR Scanner along with a 12 cm shielded gradient (BGA12) running Paravision 360.3.3 software (Bruker BioSpec, Ettlingen, Germany). A special double-tuned ^1^H/^19^F (400.2/376.8 MHz) transmit–receive surface coil (RF SUC 400 1H/19F ID = 20 L/L TR Bruker BioSpec, Ettlingen, Germany) with an inner shell diameter of 20 mm was used for both MRI and MRS measurements. To determine the contrast agent relaxivity (r1), serial dilutions of the prepared superfluorinated PPz probe were performed in PBS (pH = 7.4). Beginning with a concentration of 11.1^−3^ M, a series of dilutions was prepared in order to establish an optimal concentration range for the detection of the ^19^F MR signal. As a reference substance, PBS was used to calculate the signal-to-noise ratio (SNR) and 1 mM aqueous TFA solution was used as a positive control. Five dilutions ranging from 11.1 mM to 1.11 mM in Milli-Q water with a volume of 0.3 mL were prepared. All samples were measured in Eppendorf safe-lock polypropylene PP-tubes with a diameter of 0.5 cm. The tubes were placed in the center of a plastic box and measured at ambient temperature. For the quantitative evaluation of the SNR, regions of interest (ROIs) were placed inside each PP-tube and in the surroundings (noise). The resonance frequency and the corresponding 90-degree flip angle for the material were determined beforehand. A basic MRS spectrum was performed using an ISIS (Image Selected In vivo Spectroscopy) sequence to determine the correct resonance frequency and the AdjRefPowX procedure (part of the Paravision 360.3.3 software) was used to determine the correct reference power corresponding to a pulse with a 90-degree flip angel. Briefly, in this procedure, depending on the coil hardware (continuous wave power of the coil, in our case 1 W), radio frequency pulses of increasing length are applied and their amplitude is measured. The maximum value in a certain range is then defined as the reference power needed to achieve a flip angle of 90 degrees.

^19^F-MRI was carried out using a gradient echo fast low-angle shot (FLASH) sequence with an echo time of TE = 1.5 ms, a repetition time TR = 300 ms, a flip angle of 90 degrees and a slice thickness of 5 mm, as well as a matrix size of 32 × 32 and a field of-view (FOV) = 20 × 20 mm. Averaging over 100 scans resulted in a total acquisition time of 16 min. The sample solutions were measured in an Eppendorf vial and the ROIs were drawn according to the outline of the vials.

^19^F-MRS spectra were obtained via an image-selected in vivo spectroscopy sequence (ISIS). ISIS is recommended for short T2 nuclei in combination with the used transmit surface coil. The main parameters were TR = 2000 ms and 1024 acquisition points. Using a bandwidth of 18 kHz and a center frequency of −74 ppm resulted in an acquisition time of 5 min.

### 2.1. Synthesis of Poly(trifluoroethoxy-propargyloxy)phosphazene (TFE-Propargyl-PPz, P1)

PPz **P1** was synthesized in an argon-filled glove box according to an adapted literature procedure [26,27]. Briefly, 4-(diphenylphosphino)styrene (30.0 mg, 0.104 mmol, 1 eq.) was dissolved in ≈1 mL dry DCM and reacted with hexachloroethane (27.1 mg, 0.115 mmol, 1.1 eq.) in ≈1 mL dry DCM for 24 h. The chlorinated initiator was then added to the PPz-monomer Cl_3_PNSiMe_3_ (1.7521 g, 7.804 mmol, 75 eq) in ≈3 mL DCM and polymerization was carried out over 24 h. In parallel, 2,2,2-trifluoroethanol (1.18 mL, 15.6 mmol, 150 eq.) and propargyl alcohol (0.45 mL, 7.8 mmol, 75 eq.) were dissolved in approximately 50 mL anhydrous THF and 0.9364 g NaH (23.41 mmol, 225 eq.) was added portion-wise, avoiding excessive gas evolution and heating, and stirred in the glove box for 24 h. Afterwards, the polymer solution was added to the deprotonated alcohols in THF, resulting in a slightly turbid solution, and stirred for additional 24 h. The resulting poly(organo)phosphazene was transferred out of the glove box, the solvent was evaporated and the polymer was purified by precipitation from THF in 2x deionized water and 2x heptane. After drying in a desiccator, an off-white waxy polymer was obtained (1.336 g, 79%).

^1^H-NMR (300MHz, (CD_3_)_2_CO, δ): 3.15 (br, 44H), 4.55 (br, 196H), 4.77 (br, 94H), 5.47 (d, 1H), 6.03 (d, 1H), 6.87 (dd, 1H), 7.60–7.91 (m, 14H) ppm; ^31^P{^1^H}-NMR (121 MHz, (CD_3_)_2_CO, δ): −8.12, −7.16, −3.07, 16.64 ppm; ^19^F-NMR (282 MHz, (CD_3_)_2_CO, δ): −75.73 ppm.

### 2.2. Synthesis of Poly(trifluoroethoxy-propargyloxy-MESNa)phosphazene (TFE-MESNa-PPz, P2)

The PPz **P1** was further reacted via a thiol–ene reaction [27]. TFE-Propargyl-PPz (209.4 mg, 2.339 mmol, 1 eq.) was dissolved in 25 mL MeOH in a quartz flask and sodium 2-mercaptoethanesulfonate (656 mg, 3.92 mmol, 4 eq.) and DMPA (21 mg, 0.08 mmol, 10 wt%) were added as the photoinitiator. After degassing with Ar for 10 min, the flask was put in an UV reactor and irradiated at 365 nm at 5 °C overnight. Subsequently, the solvent was evaporated and the product dialyzed against deionized H_2_O and EtOH (1kDa MWCO) for 48 h. After freeze-drying, a white fluffy solid was obtained (173.8 mg, 41%).

^1^H-NMR (300MHz, D_2_O, δ): 2.32–3.59 (br, 16H), 4.31 (br, 18H) ppm; ^31^P{^1^H}-NMR (121 MHz, D_2_O, δ): −7.56, 2.13 ppm; ^19^F-NMR (282 MHz, D_2_O, δ): −75.81 ppm.

## 3. Results and Discussion

### 3.1. Polymer Synthesis and Characterization

**Scheme 1:**
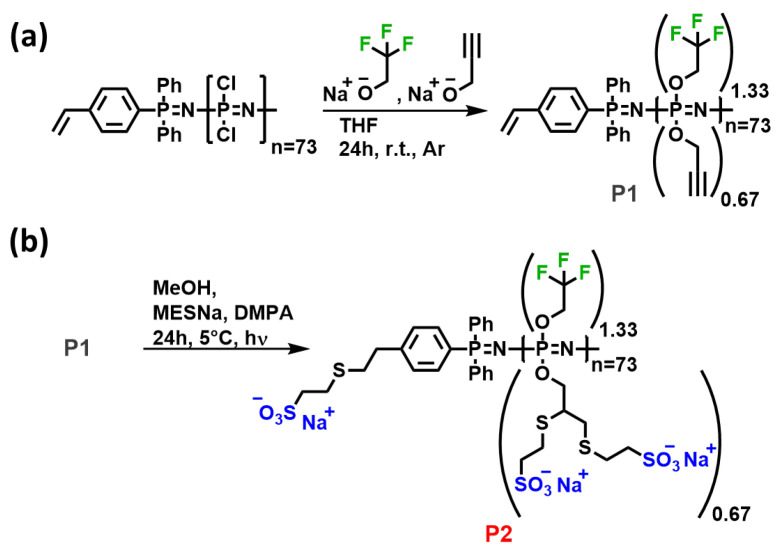
Schematic synthesis path towards polymers **P1** and **P2**. (**a**) Macromolecular substitution reaction of poly(dichloro)phosphazene yielding TFE-Propargyl-PPz (**P1**). (**b**) Thiol–yne reaction of **P1** yielding the final MRI-CA TFE-MESNa-PPz (**P2**).

A superfluorinated yet water-soluble polyphosphazene was designed to fulfil the proposed prerequisites for ideal ^19^F-MRI probes, namely a high local concentration of magnetically equivalent ^19^F-nuclei, high mobility of the fluorine nucleus, excellent aqueous solubility and biocompatibility [13]. We selected a polyphosphazene platform not only due to the extensive track record of PPz in biomedical applications but also the unique chemical constitution, [NPR_2_]_n_, of these polymers. This unique [NPR_2_]_n_ structure allows high loading, with its two functionalizable groups per repeat unit combined with an inherent high mobility of the pendant fluorine moieties. This high mobility is due to the unique structure’s ultra-low intrinsic barrier to rotation in the main chain. In fact, polydifluorophosphazene is the lowest ever calculated for skeletal bonding in polymers [33]. To realize this polymer design, a poly(dichloro)phosphazene [NPCl_2_]_n_ was synthesized via phosphine-mediated living cationic polymerization, employing 4-(diphenylphosphino)styrene as an initiator and Cl_3_PNSiMe_3_ as a monomer in a ratio 1:75, according to procedures previously developed in our laboratory [26]. Subsequently, 0.67 eq. of the pendant chlorine atoms were substituted with 2,2,2-trifluoroethanol (TFE) moieties to functionalize each phosphorus atom in the main chain at least once (Scheme 1). 

**Figure 1:**
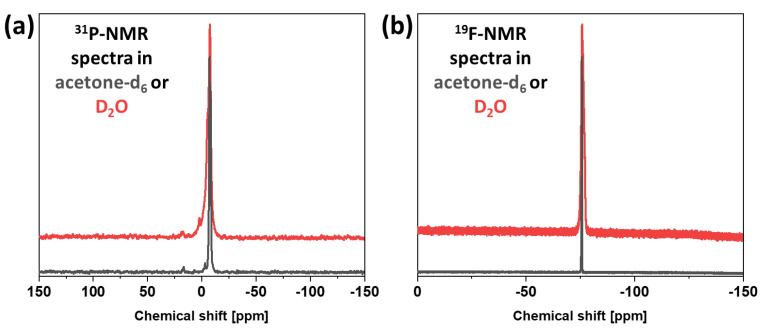
^31^P- and ^19^F-NMR-spectra of **P1** and **P2** in deuterated acetone and D_2_O, respectively. (**a**) ^31^P-NMR spectra of **P1** (black) and **P2** (red) showing a stable polyphosphazene and no change in the backbone structure after functionalization. (**b**) ^19^F-NMR spectra of **P1** (black) and **P2** (red) showing successful and stable incorporation of ^19^F-nuclei.

**Figure 2:**
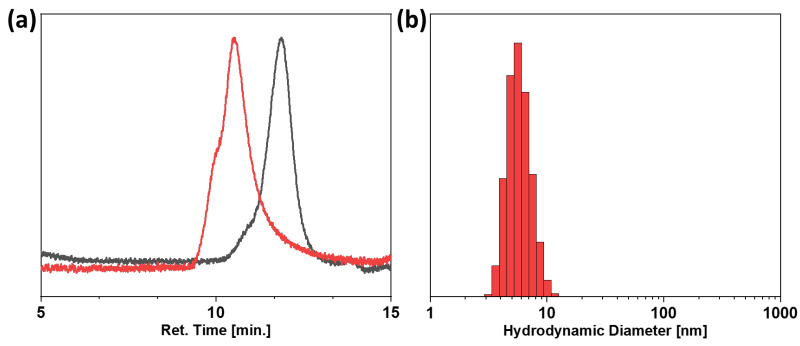
SEC and DLS measurements of TFE-Propargyl-PPz (**P1**) and TFE-MESNa-PPz (**P2**). (**a**) RI-detector traces of SEC measurements of polymers **P1** (black) and **P2** (red) in DMF. (**b**) Volume distribution of the DLS measurement of polymer **P2** in PBS pH 7.4.

Trifluoroethanol was selected to provide three identical ^19^F-nuclei per molecule, showing a single and distinct signal in ^19^F-NMR spectroscopy at around −75 ppm (Figure 1). The remaining highly labile chlorine atoms were substituted with propargyl alcohol for subsequent post-polymerization modifications yielding **P1** as a macromolecular intermediate, as shown in Scheme 1a. This intermediate was thoroughly characterized by NMR spectroscopy, showing the targeted ratio of TFE to propargyl alcohol of 2:1, as well as the approximate desired chain length with 73 repeating units as calculated from the aromatic protons of the phosphine end-group (Figure S1). As expected, the ^19^F-NMR spectrum shows the distinct peak of the trifluoroethoxy group at around −75 ppm, and the ^31^P-NMR spectrum gives the characteristic peak of the polyphosphazene backbone, demonstrating complete substitution of the backbone chlorine atoms (Figure 1a,b). In addition, the end-groups of the polymer chain were identified by ^1^H-^31^P—heteronuclear multiple bond correlation (HMBC) 2D-NMR spectroscopy Figure S7. The phosphorus signal corresponding to the α-chain end clearly correlates with the signals of the aromatic protons. The polymer was further characterized by SEC measurements in organic solvent (DMF), showing a single symmetric peak with a molecular weight of around 15 kDa and narrow dispersity of Đ = 1.1, Figure 2a.

To provide the desired water solubility of the final superfluorinated PPz, the triple bonds of the propargyl alcohol substituents were reacted with sodium 2-mercaptoethane sulfonate (MESNa) in a thiol–yne addition reaction, yielding the final TFE-MESNa-PPz **P2** (Scheme 1b). The quantitative conversion of the triple bonds was verified by FTIR spectroscopy, showing the complete disappearance of the band assigned to the triple-bond signal at around 2130 cm^−1^. Furthermore, ^1^H-NMR spectroscopy displayed the new signals of the methylene groups stemming from the triple-bond and the coupled MESNa moieties (Figures S4 and S8). Although the distinct assignment of the signals was not possible in the ^1^H-NMR spectrum due to overlapping signals and extensive peak broadening, the ratio between the signals of the substituents and the overall integrals could be assigned to the correct substitution pattern on polymer **P2**. In addition, a stable polyphosphazene backbone structure and the presence of suitable ^19^F-nuclei could be confirmed by the ^19^F- and ^31^P-NMR spectra (Figure 1a,b). SEC measurements of the sulfonated polymer showed an increase in M_n_ to around 30 kDa (Figure 2a), and DLS measurements and zeta potential analysis were performed as well. The average ζ-potential was measured at −12.5 mV in triplicate (Figure S9); however, the split signal of the triplicate measurements might indicate a slight agglomeration of the polymer at the respective concentration. This was further investigated by DLS measurements in PBS at pH 7.4, where a diameter of D_h_ = 5.9 nm according to its volume distribution could be determined (Figure 2b), further supporting the low degree of agglomeration suggested by the zeta potential analysis. The relatively small hydrodynamic diameter is to be expected, since no particulate formulation is present; only completely soluble linear polymers, as characterized by SEC. Overall, the prepared novel ^19^F-MRI CA displays only magnetically equivalent ^19^F-nuclei, a fluorine content of over 290 ^19^F-nuclei per polymer chain (17.5 wt% ^19^F on **P2**) and excellent water solubility in the range of NaCl (>360 mg mL^−1^) in PBS pH 7.4. Furthermore, they retain their high water-solubility even at lower pH with >100 mg mL^−1^ at pH 3.

### 3.2. Magnetic Resonance Imaging (MRI) and Spectroscopy (MRS)

The resonance frequency as well as the corresponding 90-degree flip angle of **P2** was calibrated for the ensuing MRI and MRS experiments. This was performed using the preprogrammed AdjRefPowX procedure, increasing the radio frequency pulse length and observing the corresponding signal amplitude. The coil and setup were tested in advance, using a cylindrical phantom containing a 1M aqueous trifluoroacetic acid (TFA) solution as a reference substance and positive control, showing a peak maximum at −76.1 ppm [34] in the ^19^F-spectra recorded by ISIS measurement. Using a sampling resolution of 89.268 μs at a pulse power of 1 W (7.071 V), the maximum was found at a pulse length of 407.243 μs (Figure S10), giving a reference power of 0.166 W to achieve a 90-degree flip angle for TFA. For **P2**, the peak maximum in the corresponding ^19^F-spectrum was recorded at −76.7 ppm according to ISIS measurements (Figure S11a). In contrast to the positive control TFA, a slightly lower sampling resolution of 77.778 μs was used to evaluate the 90-degree flip angle and the absolute maximum was found at a pulse length of 361.111 μs for a pulse power of 1 W (7.071 V), Figure S11b, giving a reference power of 0.13 W for the TFE-MESNa-PPz.

**Figure 3:**
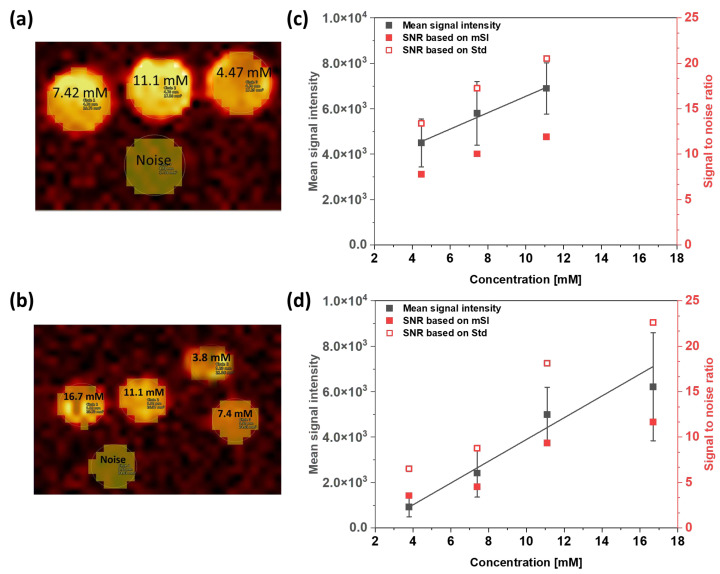
Quantitative evaluation of TFE-MESNa-PPz **P2**. (**a**,**b**) ^19^F-MRI FLASH-sequence images of **P2** in three concentrations in PBS (**a**) (4.47 mM, 7.42 mM and 11.1 mM) and in serum (**b**) (FBS, 3.8 mM, 7.4 mM, 11.1 mM and 16.7 mM). Samples are imaged in Eppendorf vials; ROIs were drawn around the axial view of the vials. A separate ROI was selected to evaluate the image noise. (**c**,**d**) Mean signal intensity (black) of the samples in PBS (**c**) and FSB (**d**) are shown in dependence of the sample concentration. The signal-to-noise ratio of each concentration is depicted, both corresponding to the mean signal intensity (mSI, full red square) and the standard deviation (Std, empty red square) of the signal intensity in the ROIs.

To evaluate the quantification of the novel contrast agent **P2**, dilution series of the TFE-MESNa-PPz in PBS pH7.4, as well as in fetal bovine serum (FBS), were prepared. ^19^F-MRI measurements were performed using a FLASH sequence for imaging, including the correct adjustment values determined above. The samples were prepared in 1 mL Eppendorf vials and the regions of interest (ROIs) drawn on the outline of the vials. As a control, the same size ROI was selected to determine the noise (Figure 3a,b). Based on these measurements, the mean signal intensity (mSI) of the ROI as well as the standard deviation (Std) were determined for every concentration and the signal-to-noise ratio (SNR) was calculated for both (Figure 3c,d). With a reasonable SNR of approximately 8 and 13 for the mSI and Std, respectively, a lower limit of 4.47 mM **P2** was required in PBS. For the dilutions in FBS, the lowest SNR evaluated was 3.5 and 6.5 for the mSI and Std of 3.8 mM TFE-MESNa-PPz. The increase in SNR correlating to the increased concentration of the TFE-MESNa-PPz can be seen in Figure 3c,d (red), showing an SNR of around 22 and 23, based on the STD, for the maximum tested concentration of 11.1 mM in PBS and 16.7 mM tested in FBS. As already stated, the signal intensity from a ^19^F MR image is directly proportional to the fluorine concentration, allowing quantitative “hot spot” MRI. Hence, a decrease in the signal intensity with the decreasing concentration of **P2** was observed. Furthermore, our results show a difference in mSI when using PBS or FBS as media. In particular, at 11.1 mM the mean signal intensity was 6864.73 for the sample in PBS and at 4984.75 for **P2** dissolved in FBS, which corresponds to a factor of approximately 1.4. At the lowest **P2** concentrations of 4.47 and 3.8 mM, in PBS and FBS, respectively, the mSI decreased approximately five times. Generally, a large number of ^19^F atoms is required in each voxel of the target volume in order to enable high conspicuity in ^19^F-MRI explaining the observed signal decrease with decreasing concentration. In addition, agglomeration with proteins present in serum can occur, which can cause differences in the chemical surroundings of the fluorine atoms, thus weakening the MRI signal as well. Although investigations regarding the relaxivities of GBCAs in different media as well as at different magnetic field strengths have been conducted [35,36], to the best of our knowledge, these are the first results evaluating the relaxivity of superfluorinated polymers in two different solvents. In accordance with the MRI measurements, the spectra recorded by MRS, using an ISIS sequence, showed an analogous increase in peak intensity with the increasing concentration of **P2** (Figure S12).

Overall, the prepared TFE-MESNa-PPz contrast agent showed high-intensity MRI signals on the phantom images (Figure 3a,b). Considering the acquisition time of about 16 min and an SNR of eight, a detection threshold of approximately 8.75 × 10^16 19^F-nuclei per voxels could be estimated for the novel ^19^F contrast agent TFE-MESNa-PPz **P2**. The same detection threshold was reported by Tirotta et al. using a superfluorinated Perfecta-based nanoemulsion as MRI probe [37], though with a significantly different acquisition time of approx. 1 h and a SNR of 3.5. Further reports on the analysis of T1 and T2 relaxation times on fluorinated samples suggest the possibility of obtaining images with high SNR using fast sequences [38,39]. Thus, our future work will not only target the systematic evaluation of the already described material by additional advanced pulse sequences available for ^19^F imaging experiments but also further development of the modular PPz-system in order to achieve shorter acquisition times together with high SNR.

## 4. Conclusions

Optimal ^19^F-MRI contrast agents are expected to provide a high number of magnetically equivalent ^19^F-nuclei to enable a sufficiently high signal-to-noise ratio, in combination with sufficient ^19^F-nuclei mobility and excellent water solubility [13,40]. While most used ^19^F-MRI agents have a fluorine content ranging from 17 fluorine atoms for perfluorooctyl bromide up to 40 fluorine atoms for perfluoropolyethers, we herein reported the synthesis of a new superfluorinated polyphosphazene ^19^F-MRI probe bearing over 290 magnetically equivalent ^19^F-nuclei per macromolecule and, hence, a fluorine content of approximately 17 wt%. Furthermore, these sulfonated derivatives of trifluoroethoxypolyphosphazene showed excellent water solubility of >360 mg mL^−1^, which is important for subsequent in vivo administration. The polymers showed a narrow dispersity of Đ = 1.1 and an average hydrodynamic diameter of 5.9 nm. With their very high fluorine content, our proposed ^19^F-MRI CAs show a single intense resonance peak in both ^19^F-MRS and MRI, avoiding any possible artefacts and misidentifications. The MRI performances and SNRs strongly indicate their exceptional capabilities as new ^19^F-MRI CAs for in vivo applications. Furthermore, due to the ease of tuneability on the PPz platform, the herein described TFE-MESNa-PPz ^19^F-MRI contrast agents hold great potential to evolve from simple hot-spot ^19^F imaging to a multifunctional platform, including multimodal imaging, diagnosis, and therapy integration.

## Data Availability

The original contributions presented in the study are included in the article/Supplementary Material, further inquiries can be directed to the corresponding author/s.

## Supplementary Materials

The following supporting information can be downloaded at https://www.mdpi.com/article/10.3390/jfb15020040/s1, Figure S1: ^1^H-NMR spectrum of TFE-Propargyl-PPz **P1** in (CD_3_)_2_CO, including peak assignment; Figure S2: ^31^P-NMR spectrum of TFE-Propargyl-PPz **P1** in (CD_3_)_2_CO; Figure S3: ^19^F-NMR spectrum of TFE-Propargyl-PPz **P1** in (CD_3_)_2_CO; Figure S4: ^1^H-NMR spectrum of TFE-MESNa-PPz **P2** in D_2_O; Figure S5: ^31^P-NMR spectrum of TFE-MESNa-PPz **P2** in D_2_O; Figure S6: ^19^F-NMR spectrum of TFE-MESNa-PPz **P2** in D_2_O; Figure S7. ^1^H-^31^P—heteronuclear multiple bond correlation (HMBC) spectrum of TFE-Propargyl-PPz **P1** in (CD_3_)_2_CO. The spectrum confirms a correlation between the aromatic protons and the phosphorus atom of the phosphine end-group at around 17 ppm (orange). Additionally, the phosphorus signal at around −7.5 ppm can be assigned to the repeating units of the polymer (violet) and the signal at around −2.8 ppm to the opposite chain end (green); Figure S8: FT-IR spectra of TFE-Propargyl-PPz **P1** and TFE-MESNa-PPz **P2** showing complete disappearance of the signal corresponding to the triple bond at around 2130 cm^−1^, highlighted in green; Figure S9: Evaluation of zeta-potential of TFE-MESNa-PPz. Measurement was carried out in triplicates (blue, black and red) and in nanopure water as a dispersant (viscosity = 0.8872 cP and refractive index, RI = 1.330) after calibration with Zeta Potential Transfer Standard (−40 mV ± 5.8 mV) showing an average ζ-potential of −12.5 mV ± 0.8 mV; Figure S10: Flip angle graph for TFA as a positive control: a) Isolated peak of **TFA** in a ^19^F-spectrum recorded via an ISIS spectroscopy sequence. b) Dependence of the signal amplitude of **TFA** at -76.1 ppm on radio frequency pulse length, as determined by the AdjRefPowX procedure, showing the maximum at a pulse length of 407.243 μs; Figure S11: Evaluation of the 90-degree flip angle of **P2**. a) Isolated peak of **P2** in a ^19^F-spectrum recorded via an ISIS spectroscopy sequence. b) Dependence of the signal amplitude of **P2** at −76.7 ppm on radio frequency pulse length, as determined by the AdjRefPowX procedure, showing the maximum at a pulse length of 361.111 μs; Figure S12: Quantitative evaluation of TFE-MESNa-PPz by MRS, based on the arbitrary peak intensity at −76.6 ppm in the respective ^19^F-spectra using an ISIS spectroscopic sequence.
