# Supplementary material for: Superfluorinated, Highly Water-Soluble Polyphosphazenes as Potential 19F Magnetic Resonance Imaging (MRI) Contrast Agents
Source: J Funct Biomater. 2024 Feb 10;15(2):40. doi: 10.3390/jfb15020040 (PMC10890119; doi:10.3390/jfb15020040)
Supplement: Supplementary file 1 [file jfb-15-00040-s001.zip › jfb-2782139-supplementary.pdf]

Supporting Information

Superfluorinated, Highly Water Soluble Polyphosphazenes as potential ^{19}F Magnetic Resonance Imaging (MRI) Contrast Agents

Paul Strasser [1], Verena Schinegger [1], Joachim Friske [2], Oliver Brüggemann [1], Thomas H. Helbich [2], Ian Teasdale [1,*] and Irena Pashkunova-Martic [2,*]

[1] Institute of Polymer Chemistry, Johannes Kepler University Linz, Altenberger Straße 69, A-4040 Linz, Austria
[2] Department of Biomedical Imaging and Image-Guided Therapy, Division of Structural and Molecular Preclinical Imaging, Medical University of Vienna and General Hospital of Vienna, 18–20 Währinger Gürtel, 1090 Vienna, Austria
* Correspondence: ian.teasdale@jku.at (I.T.); irena.pashkunova-martic@meduniwien.ac.at (I.P.-M.)

Polymer Characterization:

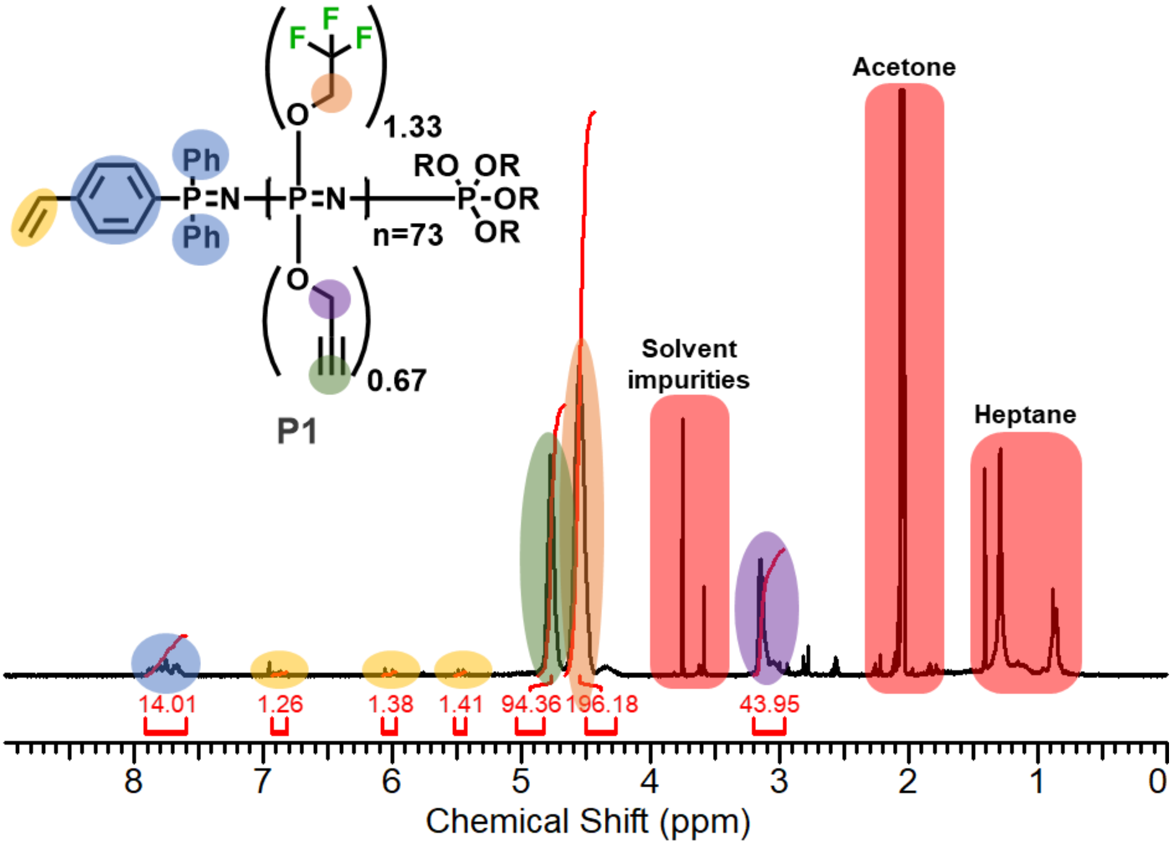

Figure S1: ^{1}H-NMR spectrum of TFE-Propargyl-PPz **P1** in $(CD_3)_2CO$, including peak assignment.

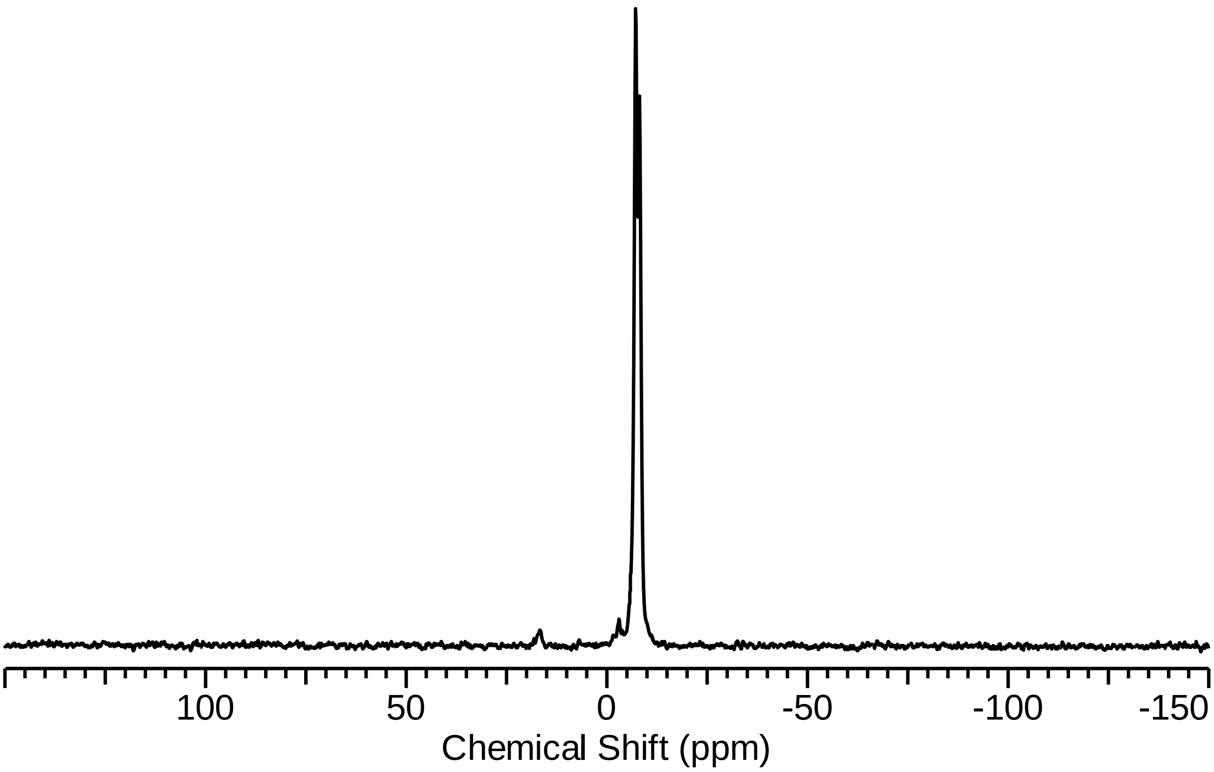

Figure S2: ^{31}P-NMR spectrum of TFE-Propargyl-PPz **P1** in $(CD_3)_2CO$.

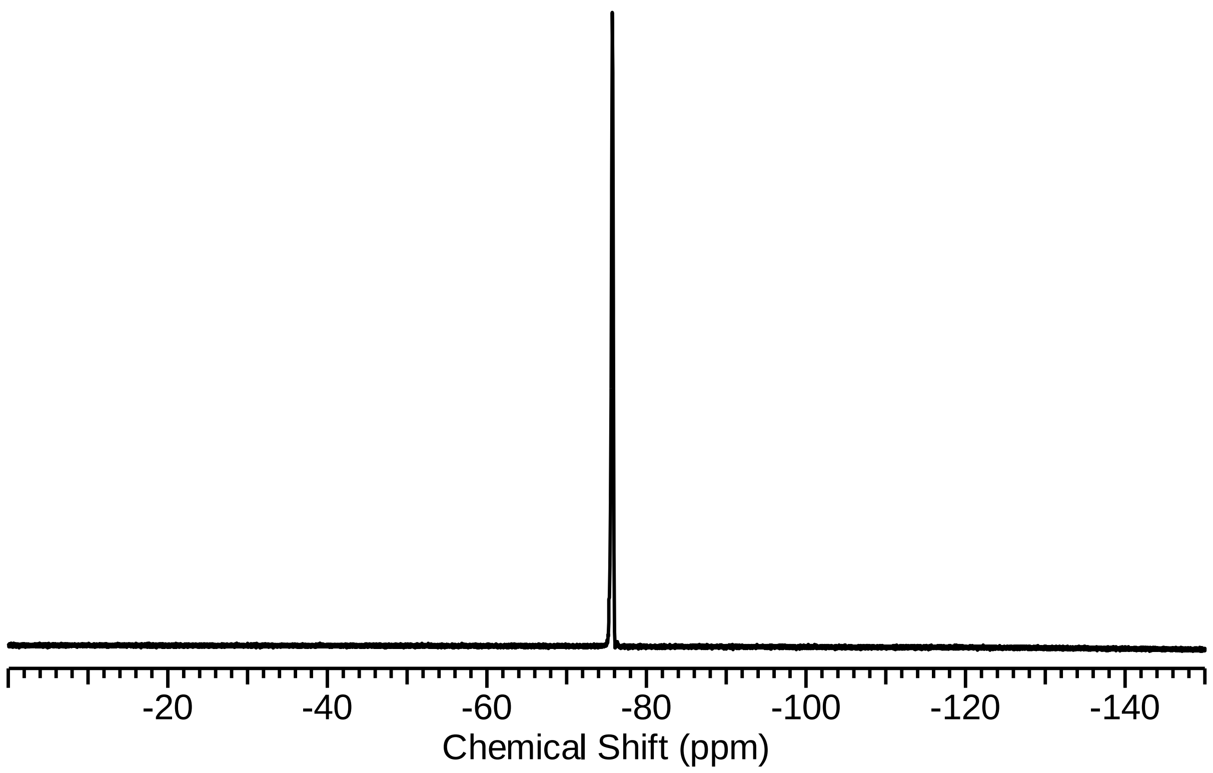

Figure S3: ^{19}F-NMR spectrum of TFE-Propargyl-PPz **P1** in $(CD_3)_2CO$.

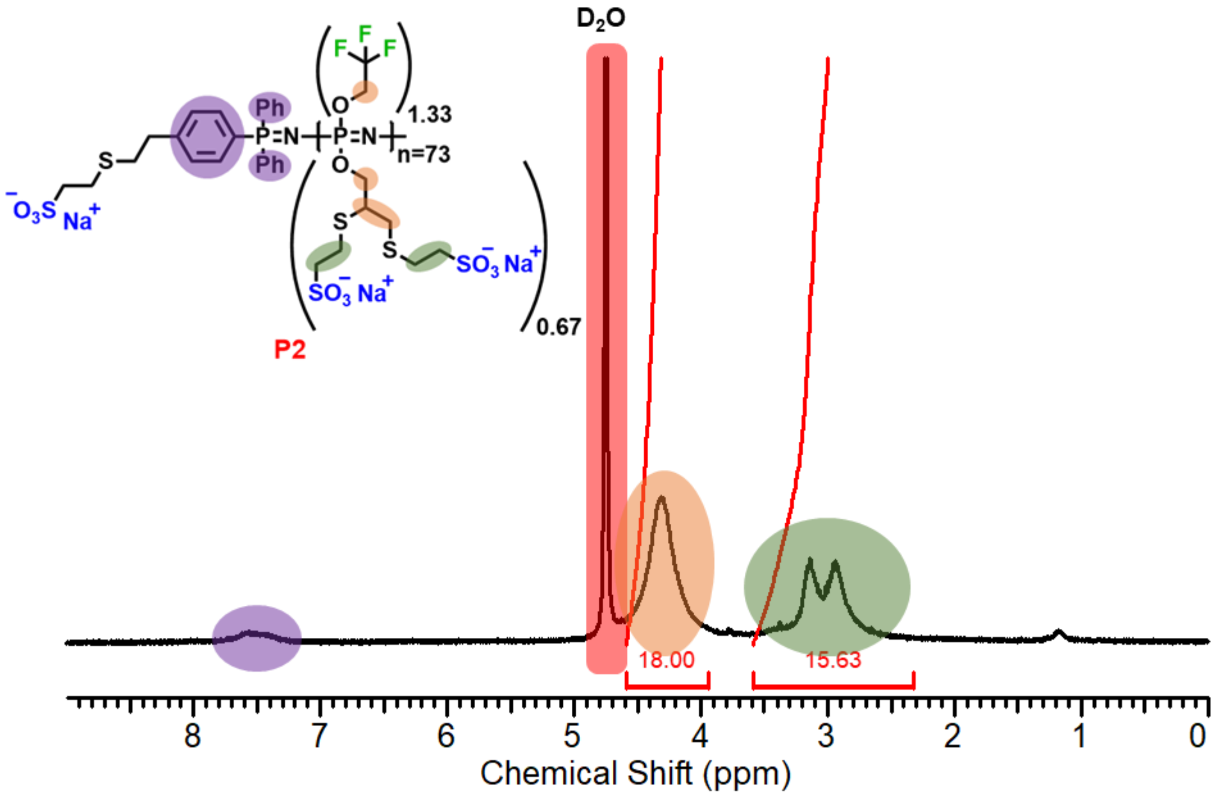

Figure S4: ^{1}H-NMR spectrum of TFE-MESNa-PPz **P2** in D_2O.

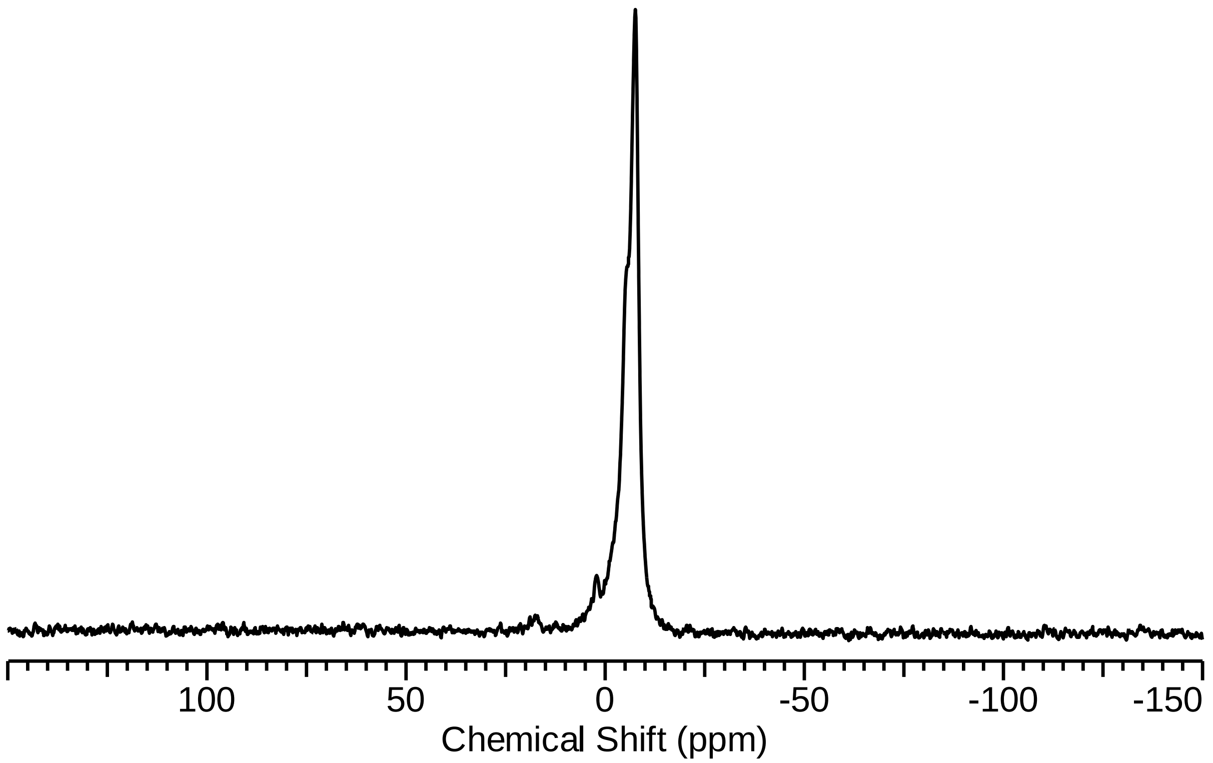

Figure S5: ^{31}P-NMR spectrum of TFE-MESNa-PPz **P2** in D_2O.

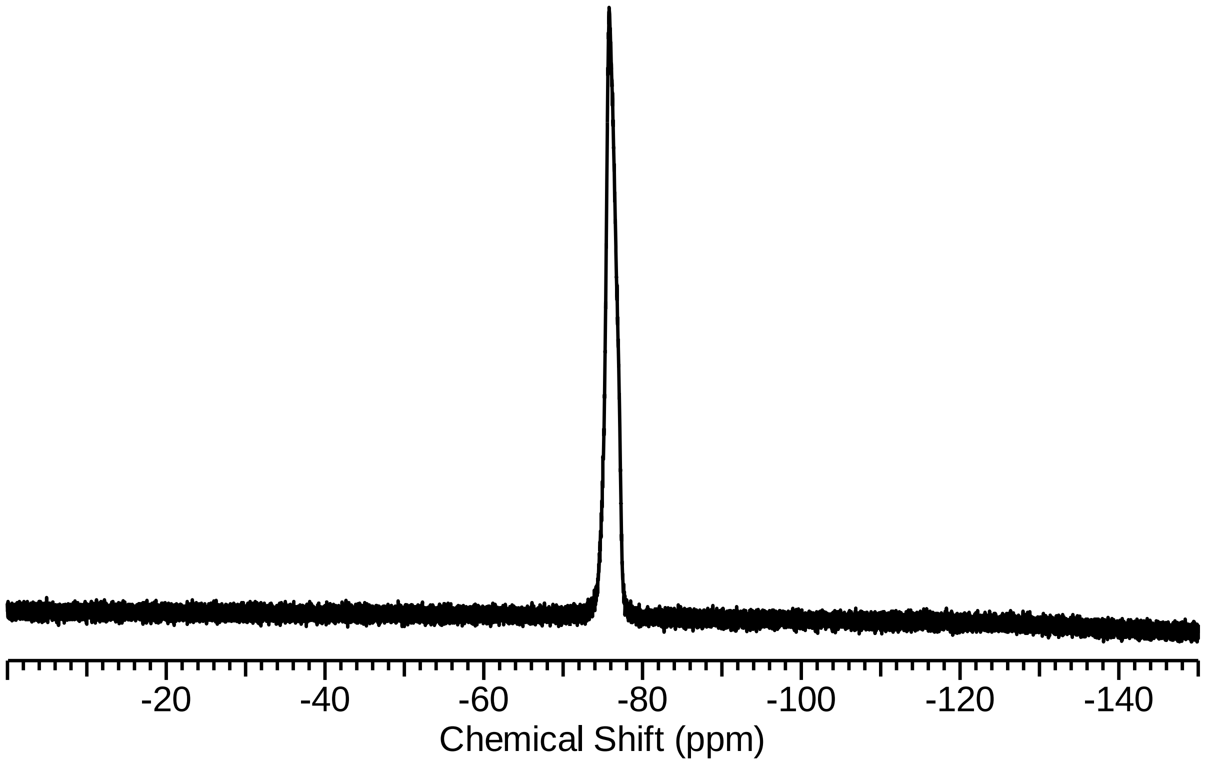

Figure S6: ^{19}F-NMR spectrum of TFE-MESNa-PPz **P2** in D_2O.

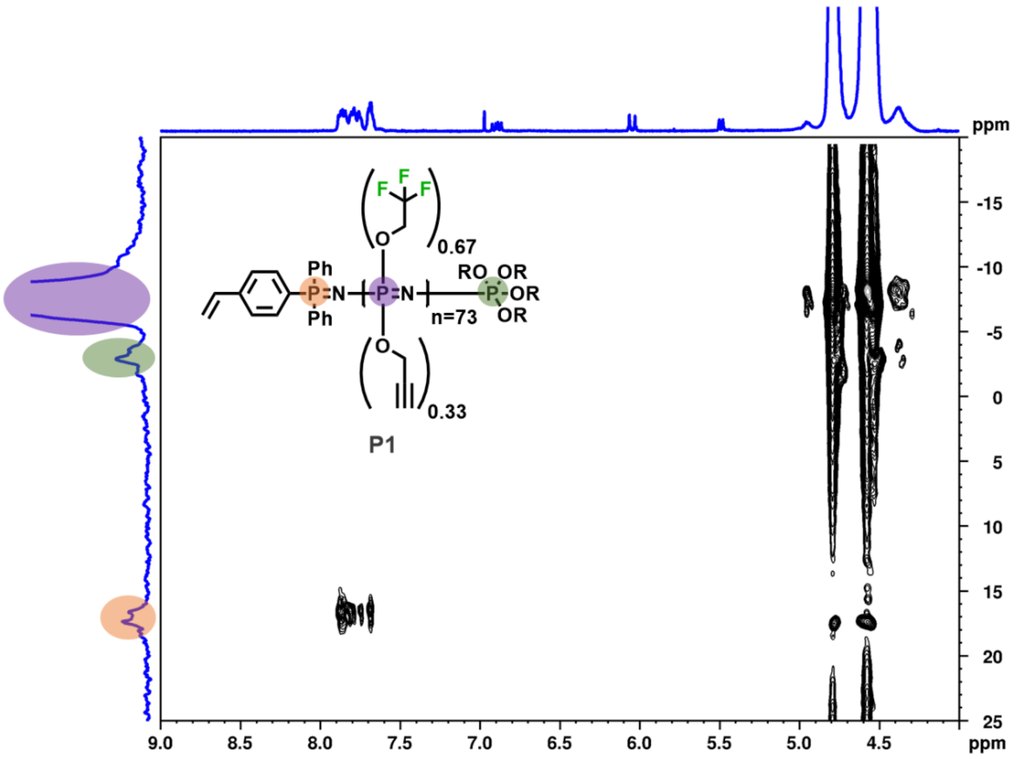

Figure S7. ^{1}H-^{31}P – heteronuclear multiple bond correlation (HMBC) spectrum of TFE-Propargyl-PPz **P1** in $(CD_3)_2CO$. The spectrum confirms a correlation between the aromatic protons and the phosphorus atom of the phosphine end-group at around 17 ppm (orange). Additionally, the phosphorus signal at around −7.5 ppm can be assigned to the repeating units of the polymer (violet) and the signal at around -2.8 ppm to the opposite chain end (green).

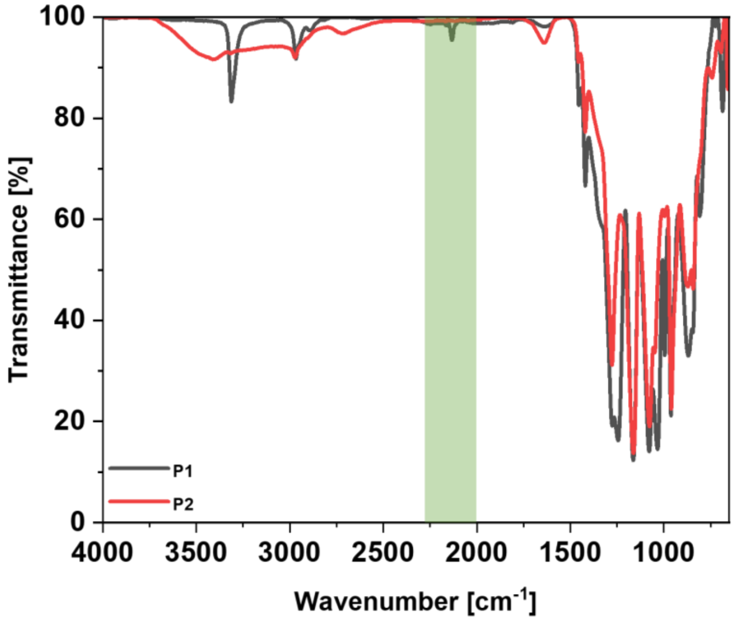

Figure S8: FT-IR spectra of TFE-Propargyl-PPz **P1** and TFE-MESNa-PPz **P2** showing complete disappearance of the signal corresponding to the triple bond at around 2130 cm^{-1}, highlighted in green.

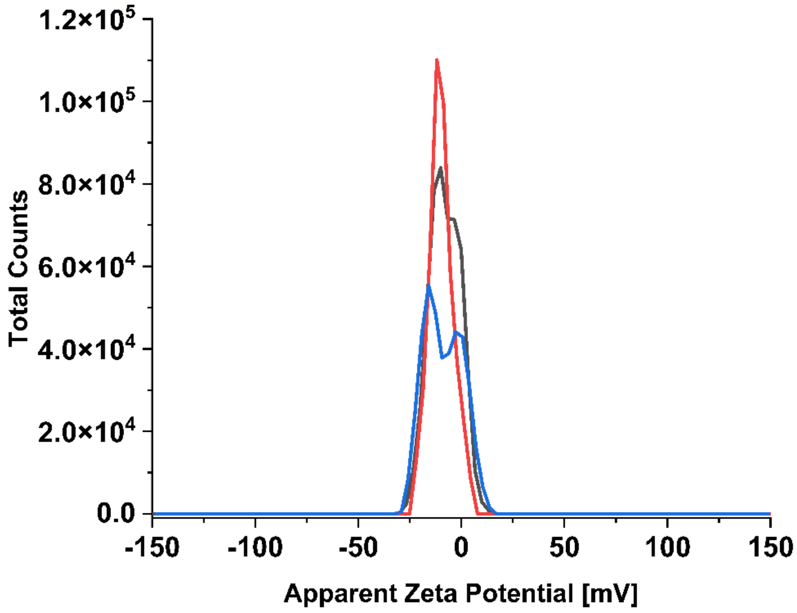

Figure S9: Evaluation of zeta-potential of TFE-MESNa-PPz. Measurement was carried out in triplicates (blue, black and red) and in nanopure water as a dispersant (viscosity = 0.8872 cP and refractive index, RI = 1.330) after calibration with Zeta Potential Transfer Standard (−40 mV ± 5.8 mV) showing an average ζ-potential of −12.5 mV ± 0.8 mV.

MRI evaluation:

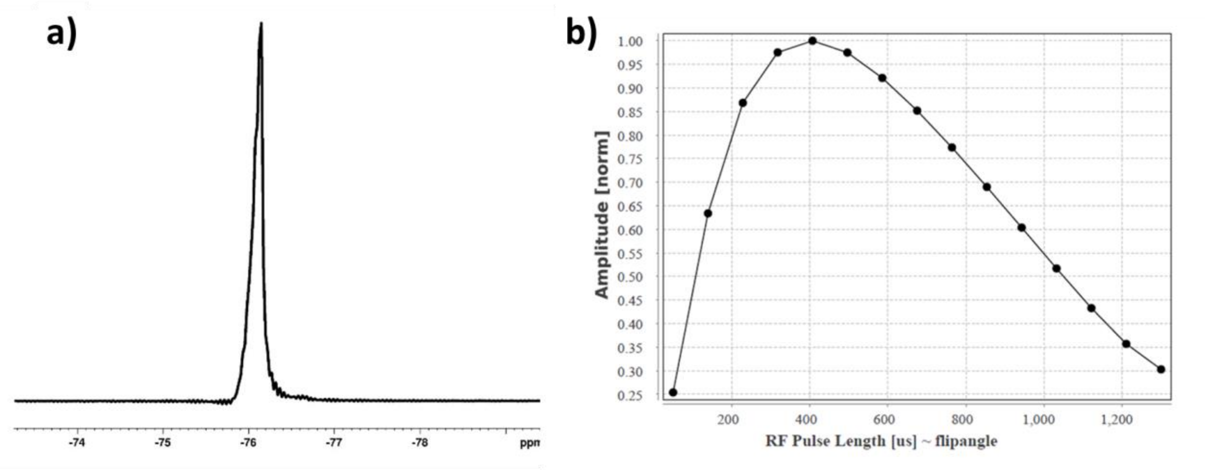

Figure S10: Flip angle graph for TFA as a positive control: a) Isolated peak of **TFA** in a ^{19}F-spectrum recorded via an ISIS spectroscopy sequence. b) Dependence of the signal amplitude of **TFA** at −76.1 ppm on radio frequency pulse length, as determined by the AdjRefPowX procedure, showing the maximum at a pulse length of 407.243 µs.

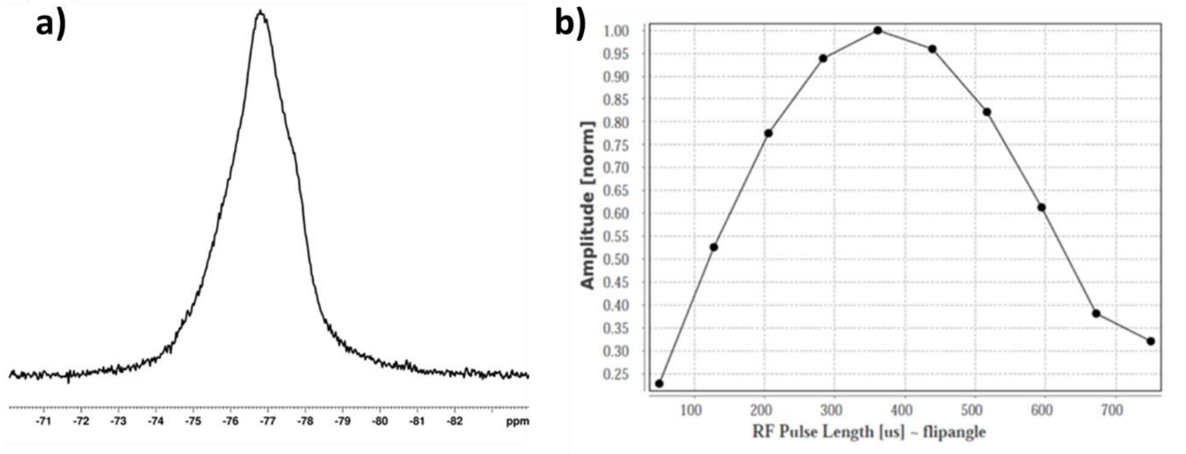

Figure S11: Evaluation of the 90-degree flip angle of **P2**. a) Isolated peak of **P2** in a ^{19}F-spectrum recorded via an ISIS spectroscopy sequence. b) Dependence of the signal amplitude of **P2** at −76.7 ppm on radio frequency pulse length, as determined by the AdjRefPowX procedure, showing the maximum at a pulse length of 361.111 µs.

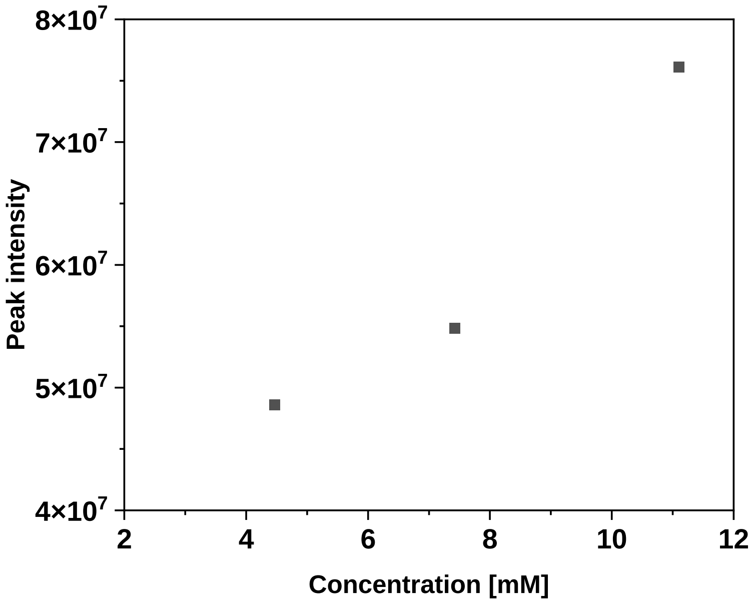

Figure S12: Quantitative evaluation of TFE-MESNa-PPz by MRS, based on the arbitrary peak intensity at −76.6 ppm in the respective ^{19}F-spectra using an ISIS spectroscopic sequence.
